# The Relationship Between Health-Related Quality of Life and Overall Survival in Patients With Advanced Renal Cell Carcinoma in CheckMate 214

**DOI:** 10.1093/oncolo/oyae003

**Published:** 2024-01-27

**Authors:** David Cella, Toni K Choueiri, Melissa Hamilton, Steven I Blum, Cristina Ivanescu, Karl Karu, Flavia Ejzykowicz, Robert J Motzer

**Affiliations:** Robert H. Lurie Comprehensive Cancer Center, Northwestern University, Chicago, IL, USA; Dana-Farber Cancer Institute, The Lank Center for Genitourinary Oncology, Boston, MA, USA; Bristol Myers Squibb, Princeton, NJ, USA; Bristol Myers Squibb, Princeton, NJ, USA; IQVIA, Amsterdam, The Netherlands; IQVIA, Tartu, Estonia; Bristol Myers Squibb, Princeton, NJ, USA; Memorial Sloan Kettering Cancer Center, New York, NY, USA

**Keywords:** health-related quality of life, patient-reported outcome measures, survival, renal cell carcinoma, prognosis, immune checkpoint inhibitors

## Abstract

**Background:**

In CheckMate 214 (median follow-up, 25.2 months), nivolumab plus ipilimumab yielded greater overall survival (OS) benefit than sunitinib in patients with intermediate-/poor-risk advanced renal cell carcinoma (aRCC). Health-related quality of life (HRQoL) assessed by the Functional Assessment of Cancer Therapy-Kidney Symptom Index-19 (FKSI-19) was also more favorable for the nivolumab plus ipilimumab group than the sunitinib group. We investigated whether HRQoL scores can predict OS of patients with 5 years follow-up in CheckMate 214.

**Patients and Methods:**

CheckMate 214 was an open-label, phase III trial in previously untreated aRCC (*N* = 1096). Patients with intermediate-/poor-risk disease (International mRCC Database Consortium prognostic score ≥ 1; *n* = 847) were randomized to either nivolumab plus ipilimumab or sunitinib monotherapy. Pooled data for OS and FKSI-19 total and subscales (disease-related symptoms [DRS], DRS-physical [DRS-P], and function/well-being [FWB]) were analyzed. Relationships between HRQoL and OS were assessed using Cox proportional hazard models with baseline and longitudinal scores. Associations between HRQoL changes and OS were assessed by landmark analyses.

**Results:**

Patients with higher FKSI-19 total and subscale scores at baseline had longer OS than patients with lower scores (HR ≤ 0.834; *P* < .0001). Longitudinal models indicated stronger associations between HRQoL and OS (HR ≤ 0.69; *P* < .001 for each). At 3 months after randomization, patients with stable/improved HRQoL versus baseline had longer median OS than patients with worsened/unobserved HRQoL versus baseline (55.9 and 26.0 months, respectively; HR = 0.56; 95% CI, 0.46-0.67; *P* < .0001). Results at 6-, 9-, and 12-month landmarks were consistent with these findings.

**Conclusion:**

In aRCC, patient-reported outcomes are important for HRQoL and prognostic evaluation.

**Clinicaltrials.gov Identifier:**

NCT02231749; https://clinicaltrials.gov/ct2/show/NCT02231749.

## Teaser

In CheckMate 214, improved overall survival (OS) and better health-related quality of life (HRQoL) were observed with nivolumab plus ipilimumab versus sunitinib in patients with previously untreated immediate-risk or poor-risk advanced renal cell carcinoma. This study analyzed 5-year follow-up data to determine whether HRQoL scores can predict the OS.

**Implications for Practice**

In CheckMate 214, improved overall survival (OS) and better health-related quality of life (HRQoL) were observed with nivolumab plus ipilimumab versus sunitinib in patients with previously untreated immediate- or poor-risk advanced renal cell carcinoma (aRCC). The present post hoc analysis of pooled data with a minimum of 5 years follow-up found that OS was longer in patients with immediate- or poor-risk aRCC who had better HRQoL scores than in those with poor HRQoL scores. While baseline HRQoL scores were associated with survival benefit, the longitudinal HRQoL scores showed a stronger association with OS. These results suggest that patient-reported outcomes, in addition to characterizing patients’ well-being and treatment experience, may have value as a predictor of clinical outcomes, such as survival.

## Introduction

Renal cell carcinoma is diagnosed at an advanced stage in approximately one-third of patients, and treatment selection and prognosis are based on established criteria for risk factors.^1,2^ Application of a validated model for prognosis assessment developed by the International Metastatic Renal Cell Carcinoma Database Consortium (IMDC) has shown that an estimated 75% of patients with advanced renal cell carcinoma (aRCC) have intermediate- or poor-risk disease, and these patients have worse clinical outcomes than patients with favorable-risk disease.^3,4^ For patients with intermediate- or poor-risk disease, clinical outcomes, including progression-free survival, have been improved with sunitinib therapy. In a phase III trial, patients in the sunitinib group had significantly longer median progression-free survival than those in the interferon alfa group.^5^ Further improvement in clinical outcomes was observed in the primary analysis of the phase III CheckMate 214 trial (median follow-up, 25.2 months), in which the first-line immunotherapy combination of nivolumab plus ipilimumab resulted in a greater overall survival (OS) benefit than sunitinib in patients with aRCC (hazard ratio [HR] = 0.63; *P* < .001).^6^ In this trial, the safety profile of nivolumab plus ipilimumab was consistent with that observed in prior studies of various types of malignancies, and the number of patients with grade 3 or 4 treatment-related adverse events was less with nivolumab plus ipilimumab than with sunitinib (250 of 547 patients [46%] vs 335 of 535 patients [63%]). Moreover, nivolumab plus ipilimumab demonstrated a higher probability of long-term OS than sunitinib in patients with intermediate- or poor-risk disease (HR = 0.68; 95% CI, 0.58-0.81; minimum follow-up, 5 years).^7^ No new safety signals were found after long-term follow-up, and the incidence of treatment-related adverse events was similar to that seen at earlier timepoints.

In addition to clinical outcomes, health-related quality of life (HRQoL) measures are important because they indicate how a disease and its treatment affect a patient’s sense of well-being and function.^8^ Because of the importance of HRQoL to patients, these measures have become an increasingly important endpoint in cancer clinical trials.^9^

In addition to assessing patients’ physical, emotional, and social well-being, HRQoL measures have been found to be predictive of OS and other clinical outcomes in RCC.^10,11^ In a previous placebo-controlled study of sorafenib for aRCC, baseline HRQoL was predictive of OS.^10^ A phase III trial of sunitinib in aRCC also determined that baseline HRQoL was predictive of time to progression or death and that better HRQoL at baseline was associated with longer progression-free survival.^11^

CheckMate 214 assessed HRQoL using the National Comprehensive Cancer/Functional Assessment of Cancer Therapy–Kidney Symptom Index-19 (FKSI-19)—a validated 19-item, kidney cancer-specific questionnaire about symptoms experienced during the previous 7 days—that patients completed.^6,7,12-14^ At a median follow-up of 25.2 months, HRQoL evaluation showed that patient-reported outcomes (PROs) were more favorable for patients with intermediate- or poor-risk disease in the nivolumab plus ipilimumab group than in the sunitinib group: the mean change in FKSI-19 total score from baseline to week 103 was an increase of 4.0 (95% CI, 1.91-6.09) for the nivolumab plus ipilimumab group and a decrease of 3.14 (95% CI, −6.03 to −0.25) for the sunitinib group (*P* < .0001).^12^

Although the primary use of PRO assessments in randomized clinical trials is to compare HRQoL between treatment arms to show treatment impact on patients, it is also of interest to identify and evaluate any relationship between HRQoL and clinical outcomes and, thus, further characterize the impact of HRQoL within a clinical trial. An initial post hoc analysis in CheckMate 214 found that OS was greater for patients with higher FKSI-19 total scores at baseline and for patients with FKSI-19 total scores that improved from baseline.^12^ Expanding on these initial findings, we report the results of a post hoc analysis using 5 years of follow-up data to further investigate the relationship between HRQoL assessed by the FKSI-19 instrument and survival benefit. Further, we report the results of landmark analyses to assess associations between changes in HRQoL and OS in patients; both pooled data and data from individual treatment groups (nivolumab plus ipilimumab vs sunitinib) were analyzed.

## Methods

### Design of CheckMate 214 and Participants

CheckMate 214 (Clinicaltrials.gov identifier: NCT02231749) has been described previously.^6^ Briefly, the study was an international, randomized, open-label, phase III trial that enrolled patients with previously untreated aRCC with a clear-cell component. Adult patients with measurable disease according to Response Evaluation Criteria in Solid Tumors, version 1.1, and a Karnofsky performance status score ≥70 were randomly assigned (1:1 ratio) in a block size of 4 to one of the following 2 treatment arms: either (1) nivolumab plus ipilimumab followed by nivolumab monotherapy or (2) sunitinib monotherapy. Stratification was based on IMDC prognostic score (0 vs 1-2 vs 3-6) and geographical region (USA vs Canada/Europe vs the rest of the world).

The institutional review board or ethics committee at each site approved CheckMate 214, which was conducted according to the Good Clinical Practice guidelines defined by the International Conference on Harmonisation. Based on the Declaration of Helsinki principles, written informed consent was provided by each patient.

### Health-Related Quality of Life

HRQoL was assessed using the FKSI-19, Functional Assessment of Cancer Therapy-General, and the 3-level version of the EQ-5D instruments^12^; because the present analysis focused on the prognostic ability of the disease-specific FKSI-19, only the results associated with the FKSI-19 were included. The FKSI-19 is a validated, RCC-specific 19-item instrument with a total score range of 0-76; higher scores indicate lower symptom burden and better HRQoL.^13,14^ In addition to the FKSI-19 total score, scores for 3 of the 5 subscales were assessed in the post hoc analysis: disease-related symptoms (DRS), DRS-physical (DRS-P), and function/well-being (FWB). The FKSI-19 disease-related symptoms-emotional subscale was not included as it consists of a single item assessing whether patients worry that their condition will get worse, and it may not fully capture the breadth of emotions that patients may be experiencing.^13^ The FKSI-19 treatment side effects (TSE) subscale was also not included because the GP5 item of this subscale is considered an assessment of overall side-effect bother, and the items for nausea and diarrhea in the TSE may be duplicated by the GP5.^13^

During CheckMate 214, the FKSI-19 was administered on days 1 and 22 during the first two 6-week cycles (cycles 1 and 2), days 1 and 29 for the next 2 cycles (cycles 3 and 4), and day 1 of all subsequent cycles (cycle 5 and beyond) while patients remained on the treatment. The FKSI-19 was also administered during safety follow-up visits after treatment discontinuation (follow-up visit 1, 30 days from the last dose ± 7 days or to coincide with the date of discontinuation [±7 days] if the date of discontinuation was >37 days after the last dose; follow-up visit 2, 84 days [±7 days] from follow-up visit 1).^6,12^

### Statistical Analysis

In this post hoc exploratory analysis, we investigated the relationship between OS and FKSI-19 scores, irrespective of treatment. We focused on the FKSI-19 total score and the DRS, DRS-P, and FWB subscales. Analyses were conducted on the intermediate- and poor-risk disease group in the full-analysis set, defined as all patients with an IMDC prognostic score ≥1 at the time of randomization who were randomized to any treatment arm. This group is consistent with the primary efficacy population and the approved indication for nivolumab plus ipilimumab. Data from both treatment arms (nivolumab plus ipilimumab/nivolumab and sunitinib) groups were both pooled and analyzed individually. Analyses were not adjusted for multiplicity.

To model the relationship between HRQoL scores and OS, 2 Cox proportional hazards models were used. The first model was used to assess the association between baseline scores (ie, time invariant) and OS, whereas the second model was used to assess whether longitudinal changes in scores (ie, time dependent) were associated with better OS. In addition to the baseline and time-dependent HRQoL scores, each model also included treatment arm (categorical [nivolumab plus ipilimumab vs sunitinib]), IMDC prognostic score (1-2 vs 3-6), and geographic region (USA vs Canada and Western/Northern Europe vs the rest of the world) as covariates. OS was defined as the time from randomization to death due to any cause. Patients who did not die within 12 weeks of their last PRO assessment were censored in the longitudinal model. Patients without a baseline PRO value were excluded from the analysis. Separate models were fitted for each HRQoL scale.

The HR with associated 95% CIs for the HRQoL variable was the key measure and was calculated as the hazard of death per 𝑥 points improvement, with 𝑥 defined as established thresholds for meaningful change, where available, or was derived using distribution-based approach in the study. Specifically, the following values were used: FKSI-19 total score was 5 points; for DRS, 3 points^15^; for DRS-P, 4 points; and for FWB, 3 points.

The association between change in HRQoL and OS was also assessed by means of a landmark analysis. A landmark analysis takes a particular landmark timepoint and reruns the time-to-event analysis (eg, a Cox proportional hazards model), excluding any events that occurred before the landmark timepoint in the analysis.^16^ In the present analysis, landmarks at 3, 6, 9, and 12 months after randomization were used. Patients were classified as those with stable or improved HRQoL and those with worsened or unobserved HRQoL. Stable HRQoL was defined as scores that did not meaningfully worsen or improve compared with baseline, improved HRQoL was defined as scores that were increased by at least the meaningful change threshold compared with baseline, worsened HRQoL was defined as scores that were reduced by at least the meaningful change threshold compared with baseline, and unobserved HRQoL scores were defined as missing scores. Patients with unobserved HRQoL scores were included to ensure a conservative approach. For each landmark, a separate Cox proportional hazard regression model was fitted for the FKSI-19 total score and DRS-P, DRS, and FWB subscales. The covariates in the model were the baseline HRQoL score, HRQoL response status at the landmark, treatment arm (categorical [nivolumab plus ipilimumab vs sunitinib]), IMDC prognostic score (1-2 vs 3-6), and geographic region (USA vs Canada/western Europe/northern Europe vs rest of the world). HR and 95% CIs for survival after the landmark were calculated by using the Efron method of tie handling.

Kaplan-Meier curves were used to estimate distributions for each of the scores at each landmark. The *P*-values are from a chi-squared test for the HRQoL response parameter estimated in the proportional hazards model.

Given that the nature of the study was exploratory and that several hypotheses about HRQoL measures were individually explored, issues with multiplicity did not arise with regard to the individual hypotheses. Conclusions regarding the significance of association for individual HRQoL measures refer to the nominal alpha level for the statistical test (ie, a *P-*value of <.05 has a 5% probability of being due to chance). Data were analyzed using SAS, version 9.4 (SAS Institute, Cary, NC).

## Results

### Patient Characteristics at Baseline

A total of 847 patients with intermediate- and poor-risk aRCC participated in CheckMate 214. Most patients were male (73%), <65 years of age (62%), and had an intermediate IMDC prognostic risk (79%; Table 1). The mean FKSI-19 total score at baseline was 59.6, which is similar to the mean score for a sample of the general adult US population (59.8).^17^ At baseline, a high proportion of patients (≥95%) completed the FKSI-19 questionnaire,^12^ and these completion rates remained relatively high through week 307 for available patients in the nivolumab plus ipilimumab arm (≥75% at each timepoint during treatment except week 295 [71.4%]) as well as the sunitinib monotherapy arm (≥75% at each timepoint during treatment except week 205 [66.7%]).

**Table 1.** Patient characteristics at baseline in CheckMate 214.

Patients with IMDC intermediate- and poor-risk RCC	Total (*N* = 847)
Age, median (range), years	61 (21-85)
Age category	
<65 years	524 (61.9)
≥65 years	323 (38.1)
Sex, *n* (%)	
Male	615 (72.6)
Female	232 (27.4)
Geographic region, *n* (%)	
United States	223 (26.3)
Canada and Europe	294 (34.7)
Rest of the world	330 (39.0)
IMDC prognostic risk, *n* (%)	
Intermediate	667 (79)
Poor	180 (21)
Quantifiable tumor PD-L1 expression	
<1%	562 (66.4)
≥1%	214 (25.3)
Not reported	71 (8.4)
Karnofsky performance status	
≥90	581 (68.6)
<90	266 (31.4)
LDH level	
≤1.5 × ULN	787 (92.9)
>1.5 × ULN	45 (5.3)
Not reported	15 (1.8)
Prior radiotherapy	
Yes	104 (12.3)
No	743 (87.7)
Prior nephrectomy	
Yes	660 (77.9)
No	187 (22.1)
Time from initial diagnosis to randomization	
<1 year	590 (69.7)
≥1 year	257 (30.3)
FKSI-19 scores	
Total score (*n* = 813)	59.6
FKSI-19 DRS (*n* = 814)	30.4
FKSI-19 DRS-P (*n* = 815)	38.6
FKSI-19 FWB (*n* = 814)	7.6

Abbreviations: DRS, disease-related symptoms; DRS-P, disease-related symptoms-physical; FKSI-19, Functional Assessment of Cancer Therapy-Kidney Symptom Index-19; FWB, function/well-being; IMDC, International Metastatic Renal Cell Carcinoma Database Consortium; LDH, lactate dehydrogenase; PD-L1, programmed cell death ligand 1; RCC, renal cell carcinoma; ULN, upper limit of normal.

### Association Between Baseline HRQoL Scores and OS

**Figure 1.**
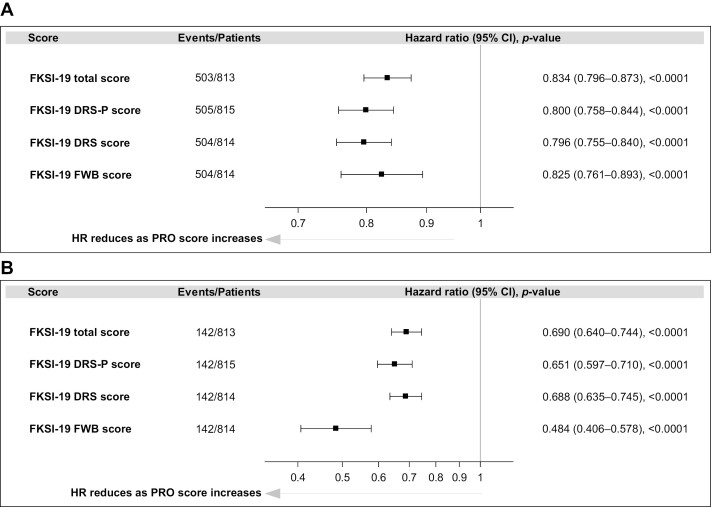
Association between OS and PRO scores in CheckMate 214 (patients with intermediate- or poor-risk RCC [all randomized])^a,b^ (**A**) Baseline model. (**B**) Longitudinal model. ^a^HR was calculated as risk of death per 𝑥-point increase from the baseline HRQoL score; 𝑥 is defined as 5 points for FKSI-19 total score, 3 points for DRS, 4 points for DRS-P, and 3 points for FWB. ^b^A lower HR indicates a stronger association between a higher HRQoL baseline score and a lower risk of death.

At baseline, FKSI-19 total scores were available for 813 patients. The baseline model demonstrated that baseline HRQoL scores are predictive of OS. During the 5-year follow-up, patients with higher FKSI-19 total scores at baseline had a 16.6% reduction in risk of death (HR = 0.834; 95% CI, 0.796-0.873) than did patients with lower FSKI-19 total scores at baseline (Fig. 1A). This reduced HR in relation to higher HRQoL scores at baseline was also observed when models for DRS-P (20.0% reduction), DRS (20.4%), and FWB (17.5%) were evaluated (Fig. 1A; HR < 1 and *P* < .0001 for each).

### Association Between Longitudinal HRQoL Scores and OS

A strong association was observed between HRQoL and risk of death during treatment in the longitudinal model (Fig. 1B). Based on the analyses of the FKSI-19 total scores, every 5-point improvement in total score was associated with a 31% reduction in risk of death (HR = 0.690; 95% CI, 0.640-0.744). The largest reduction in risk of death was associated with improvement in the FWB subscale, for which every 3-point improvement in FWB score was associated with a 52% reduction in risk of death (HR = 0.484; 95% CI, 0.406-0.578).

### Landmark Analyses

**Figure 2.**
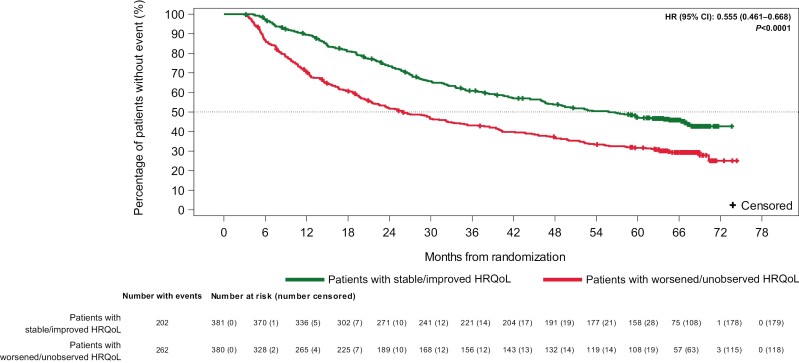
Kaplan-Meier plot of OS by response in FKSI-19. Total score at the 3-month landmark (overall [threshold = 5]): patients with intermediate- or poor-risk RCC (all randomized)^a^. ^a^HR is relative to patients with a worsening or unobserved HRQoL response, with HR < 1 favoring patients with stable or improved HRQoL. HR is derived from a stratified Cox regression model with response as the only covariate and strata as the randomization factors. The *P*-value corresponds to the Cox regression model.

Landmark analyses of the pooled data from both treatment arms supported the results from the Cox proportional hazards models and showed that patients with stable or improved HRQoL compared with baseline had a lower risk of death at each landmark than those with worsened or unobserved HRQoL compared with baseline. At the landmark of 3 months, the median OS was 55.9 months for patients with stable or improved HRQoL and 26.0 months for those with worsened or unobserved HRQoL (HR = 0.56; 95% CI, 0.46-0.67; *P* < .0001; Fig. 2). Results at the 6-, 9-, and 12-month landmarks were consistent with those at the 3-month landmark (Supplementary Fig. S1).

**Figure 3.**
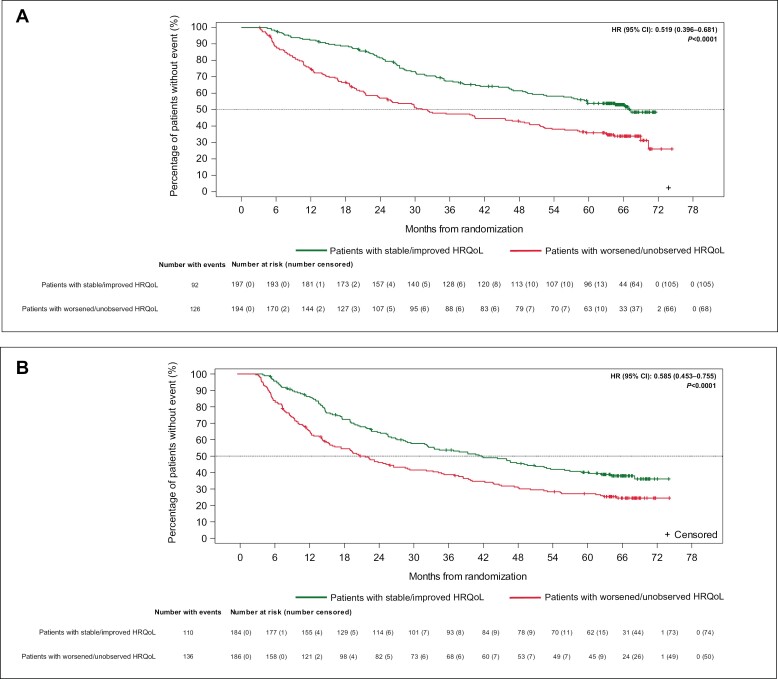
Kaplan-Meier plots of OS by response in FKSI-19 total score at the 3-month landmark for each treatment group (threshold = 5): patients with intermediate- or poor-risk RCC (all randomized)^a^. (**A**) Nivolumab + ipilimumab. (**B**) Sunitinib alone. ^a^HR is relative to patients with a worsening or unobserved HRQoL response, with HR < 1 favoring patients with stable or improved HRQoL. HR is derived from a stratified Cox regression model with response as the only covariate and strata as the randomization factors. The *P-*value corresponds to the Cox regression model.

The improvement in median OS was observed regardless of individual treatment group. At the 3-month landmark in the nivolumab + ipilimumab (Fig. 3A) and sunitinib (Fig. 3B) groups, better median OS was found for patients with stable or improved HRQoL compared with baseline than for those with HRQoL that worsened from baseline or was unobserved. Results for patients with stable or improved HRQoL at the 3-month landmark were comparable to those observed at the landmarks of 6, 9, and 12 months in each treatment group (Supplementary Fig. S2), but the HRs for the nivolumab plus ipilimumab groups indicated a greater reduction in the risk of death at the 6-, 9-, and 12-month landmarks (Supplementary Fig. S2A, S2C, S2E). In addition, patients with stable or improved HRQoL in the nivolumab plus ipilimumab group had not reached median OS by the 9- and 12-month landmarks (Supplementary Fig. S2C, S2E).

## Discussion

Overall, the results from our analyses in CheckMate 214 demonstrate a strong association between HRQoL and OS in patients with intermediate- or poor-risk aRCC irrespective of treatment (minimum follow-up of 5 years). Although the association at baseline was strong, the time-dependent (longitudinal) association was even stronger; for example, for every 5-point improvement in FKSI-19 total score, there was a 31% reduction in the risk of death in the longitudinal model versus a 17% reduction in the baseline model. In addition, these associations were apparent for the DRS-P, DRS, and FWB subscales. Lastly, irrespective of whether pooled data from treatment groups were analyzed or whether data from individual treatment groups were analyzed, patients with stable or improved HRQoL from baseline had longer median OS than did patients with worsened or unobserved HRQoL.

The current findings from the models using baseline and longitudinal HRQoL are consistent with those from a previous post hoc analysis of the association between FKSI-19 total scores at baseline, change in FKSI-19 total scores up to 25 weeks, and OS in CheckMate 214 (*n* = 847).^12^ In this previous analysis, high-baseline scores were those at or above the median and low-baseline scores were below the median; the changes in scores were described as “improved” or “not improved” (defined as those with no 3-point or greater change from baseline in FKSI-19 total score). The longest OS estimates were for patients with high FKSI-19 total scores at baseline and improved total scores longitudinally, and for those with high FKSI-19 total scores at baseline and no improvement in total scores longitudinally. The median OS was not reached for these 2 groups. Patients with low FKSI-19 total scores at baseline had the lowest OS estimates, regardless of improvement in total scores longitudinally (those with improvement: median OS: 25.7 months, 95% CI, 22.2-not estimable; those with no improvement: median OS: 16.6 months, 95% CI, 12.4-19.9).

The present analyses were not designed to detect a causal relationship between HRQoL and OS. Based on our results showing an association between the 2, we hypothesize that identification of HRQoL factors associated with survival could inform modification of treatment regimens to improve HRQoL and thus, possibly, OS in patients with low or deteriorating HRQoL. The prognostic value of HRQoL may reflect patients’ experiences beyond those evaluated by conventional techniques (eg, clinical characteristics). Functioning and well-being related to survival may be captured by PRO assessments but not by clinical prognostic indicators.^18,19^ Other clinical parameters may reflect disease activity or progression, but they may not always be accompanied by a change in the functioning or well-being of the patient.

Compared with performance status and other measures, HRQoL may be more sensitive to changes in patients’ well-being related to prognosis. For example, the Eastern Cooperative Oncology Group Performance Status scale is a single item that assesses patient self-care, daily activities, and physical ability or disability, whereas the FKSI-19 is more focused on DRS with several items that assess function and, it is completed directly by the patient without any interpretation from others.^12,20^ The FKSI-19 assesses both symptom burden and FWB, which includes the ability to work and enjoy life and overall quality of life.^13,14^ In addition, HRQoL may be affected by challenges related to the disease—patients who are skilled to deal with such issues may have better HRQoL.^19^

The strengths of the study include the large sample analyzed. Of the 1096 patients randomized in CheckMate 214, 847 had intermediate- or poor-risk aRCC, and pooled data from the 2 treatment groups were analyzed in the exploratory analyses.^6^ Another strength is the use of a validated instrument to assess HRQoL. The FKSI-19, the basis of our analyses, is a kidney cancer-specific instrument that has been extensively used in clinical trials for RCC and is based on symptoms considered the highest priority by patients with aRCC and by experienced clinicians.^13,21^ In addition, large proportions of available patients completed the FKSI-19 questionnaire throughout CheckMate 214 at baseline (≥95% of patients),^12^ at approximately 4 years of follow-up, and later (67% of patients in the sunitinib arm at week 205 and 71% in the nivolumab + ipilimumab arm at week 295); however, patients who discontinued the trial were not included. Because of the large sample size, the use of pooled data from the treatment groups, use of a validated PRO questionnaire, and the large proportion of eligible patients completing the questionnaires during a long follow-up period, the time-to-event data used in our analyses were mature and reliable.

Our analyses have some limitations. Selection bias resulting from clinical trial recruitment may have occurred; thus, the patient sample may not be representative of all patients with aRCC. Additionally, fewer FKSI-19 questionnaires were completed after disease progression and treatment discontinuation; patients who discontinued treatment had only 2 additional follow-up visits during which the FKSI-19 was collected.^12^

## Conclusions

At a minimum follow-up of 5 years, better HRQoL as determined from the FKSI-19 total score and 3 FKSI-19 subscales were associated with increased survival in patients with intermediate-/poor-risk aRCC treated in CheckMate 214; findings from the present analysis are consistent with initial results, which showed that, compared with sunitinib, nivolumab plus ipilimumab resulted in better HRQoL in this patient population (median follow-up, 25.2 months).^12^ The reductions in the risk of death associated with longitudinal HRQoL scores (minimum follow-up, 5 years) were greater than those seen with baseline HRQoL scores. In addition, patients with stable or improved HRQoL had a decreased risk of death compared with those with worsened or unobserved HRQoL, irrespective of treatment. Collectively, the results show that PROs, in addition to measuring humanistic outcomes such as how patients feel and function, may also be predictors of clinical outcomes, such as survival.

## Supplementary Material

oyae003_suppl_Supplementary_Figure_S1

oyae003_suppl_Supplementary_Figure_S2

oyae003_suppl_Supplementary_Figure_Captions

## Data Availability

Bristol Myers Squibb’s policy on data sharing may be found online (see https://www.bms.com/researchers-and-partners/independent-research/data-sharing-request-process.html).
