# Supplementary figures and images for: The Relationship Between Health-Related Quality of Life and Overall Survival in Patients With Advanced Renal Cell Carcinoma in CheckMate 214
Source: Oncologist. 2024 Jan 27;29(6):511–8. doi: 10.1093/oncolo/oyae003 (PMC11144972; doi:10.1093/oncolo/oyae003)

Figure S1A

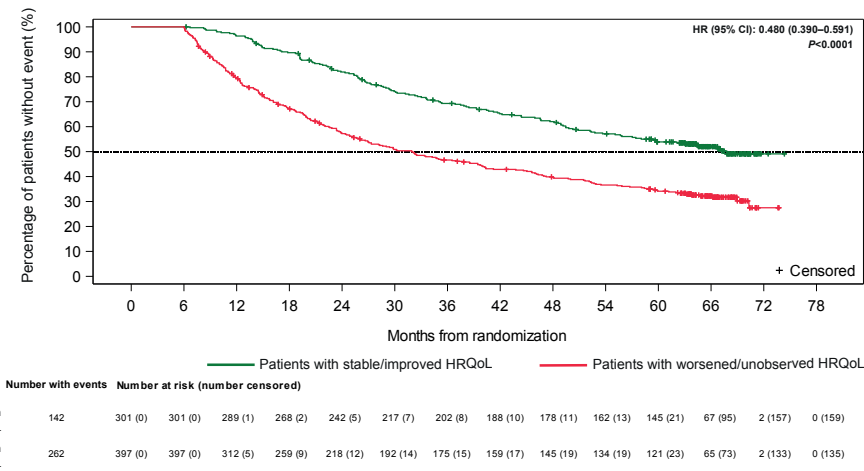

Figure S1B

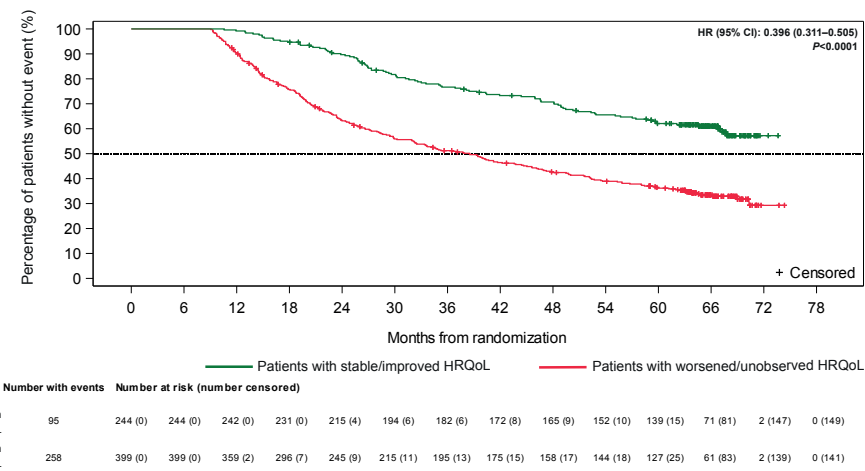

Figure S1C

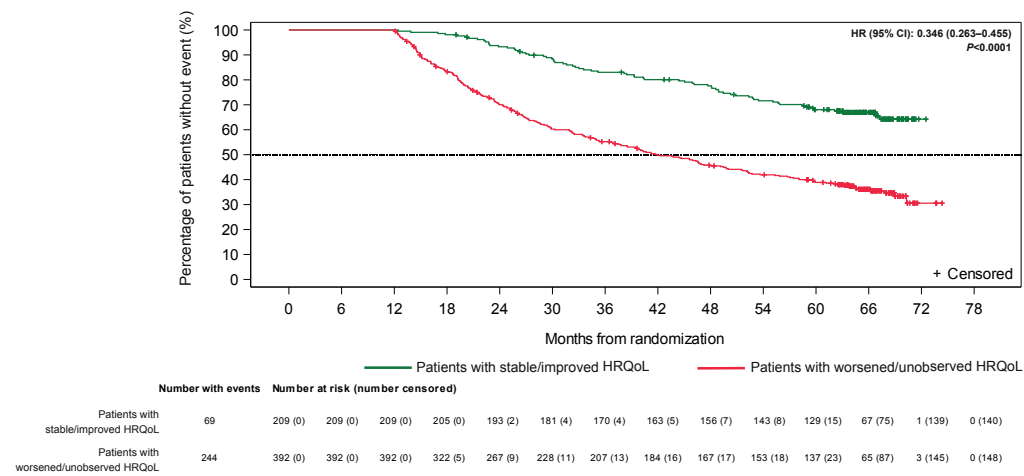

Supplement: oyae003_suppl_Supplementary_Figure_S1 [file oyae003_suppl_supplementary_figure_s1.pdf]

Figure S2A

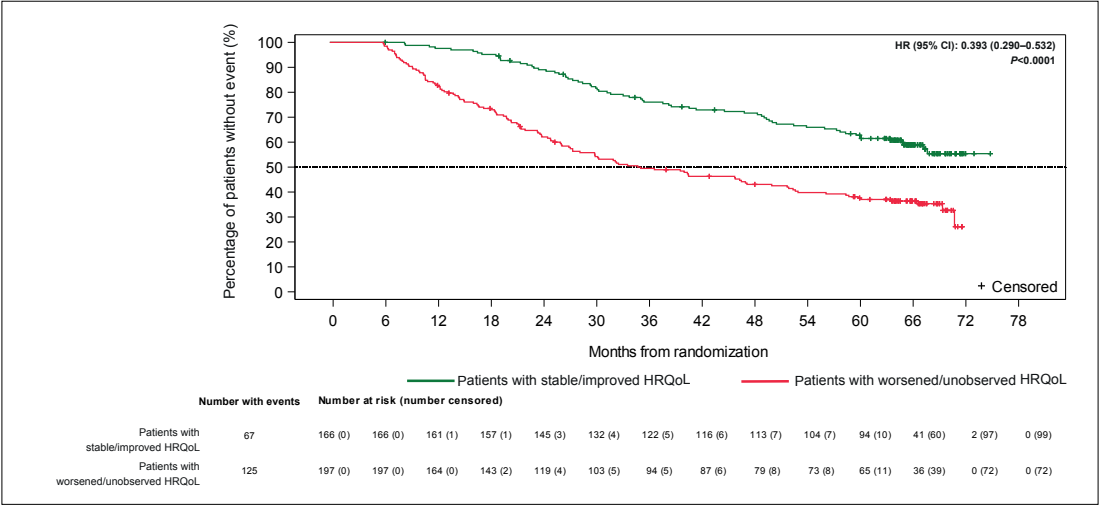

Figure S2B

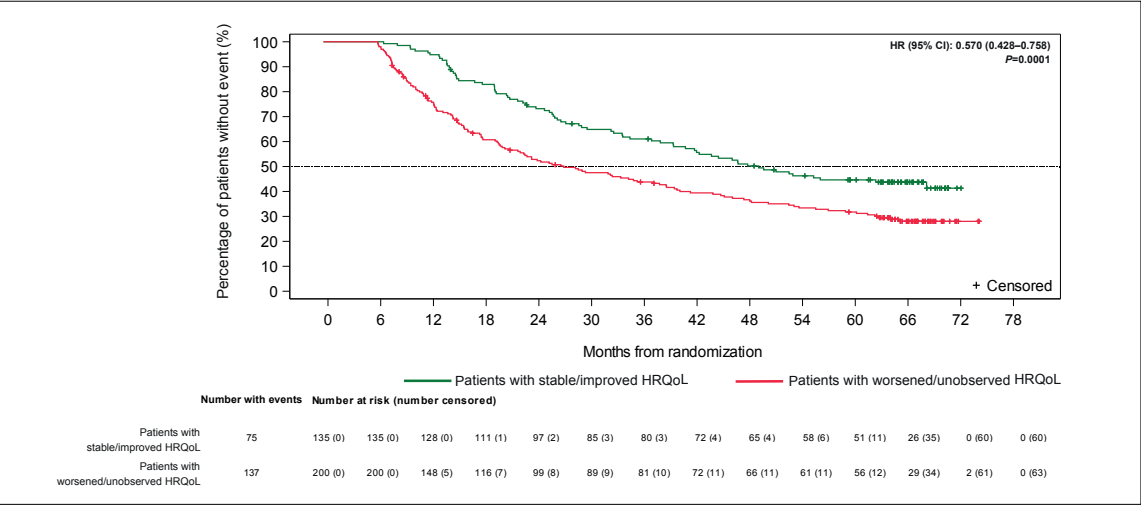

Figure S2C

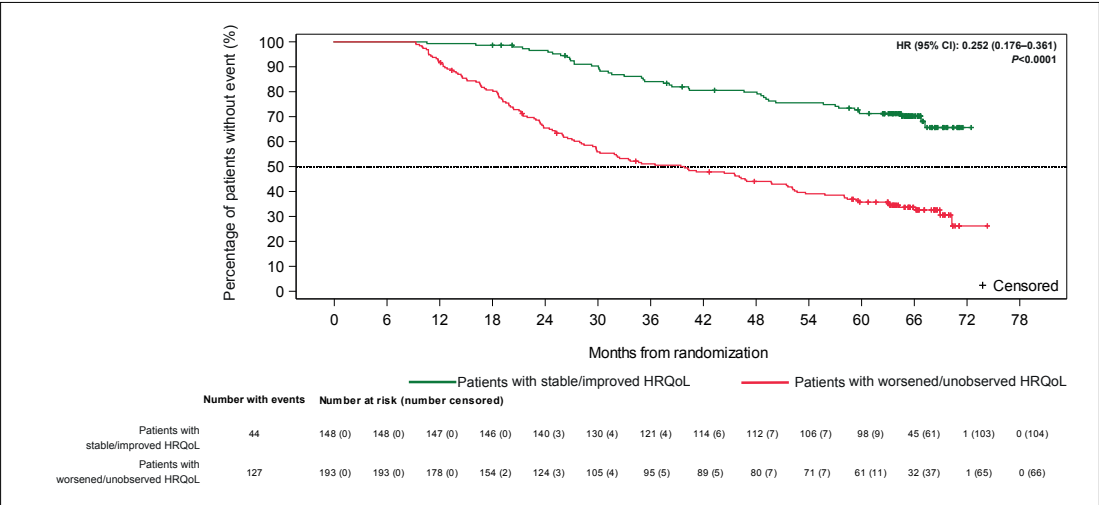

Figure S2D

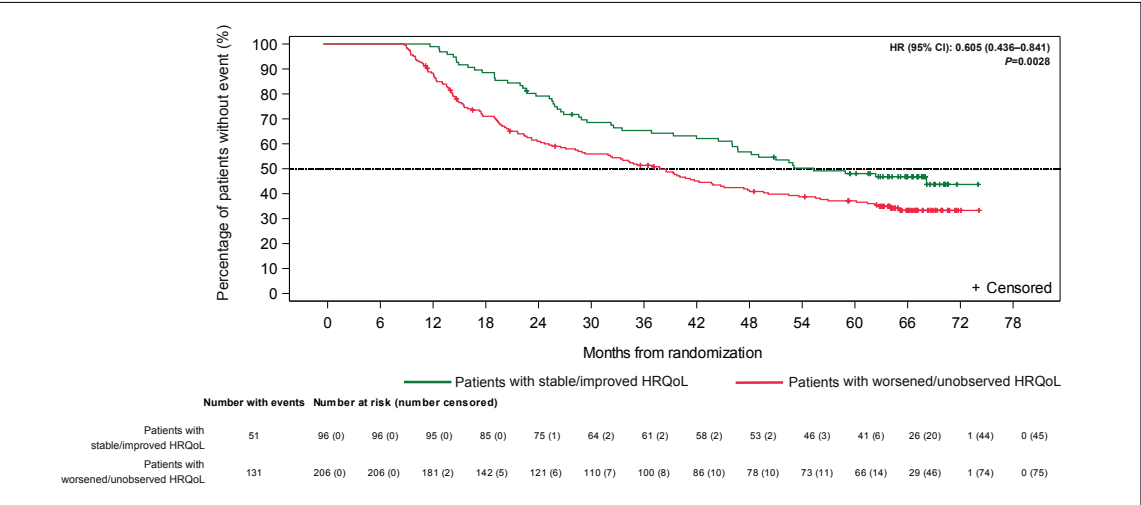

Figure S2E

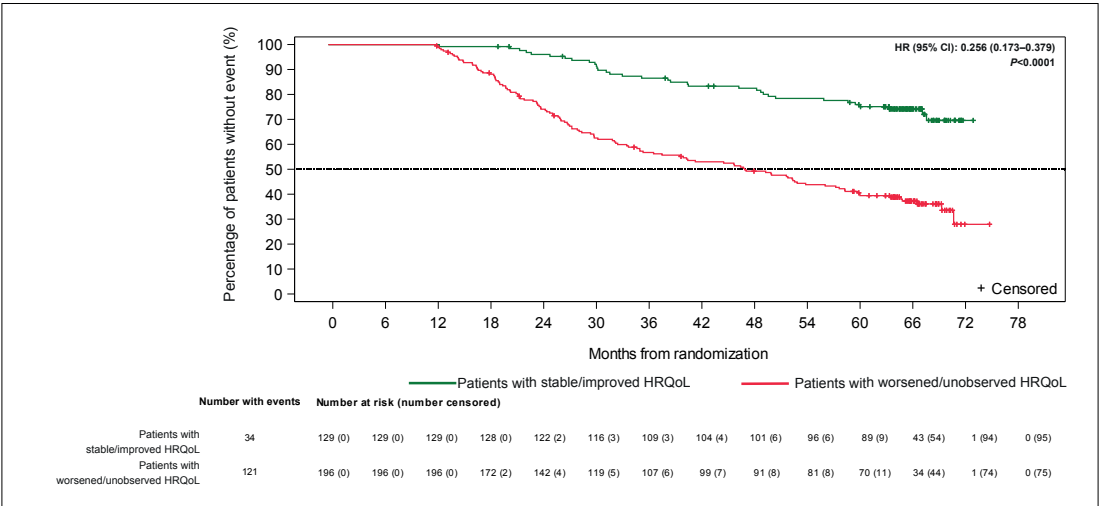

Figure S2F

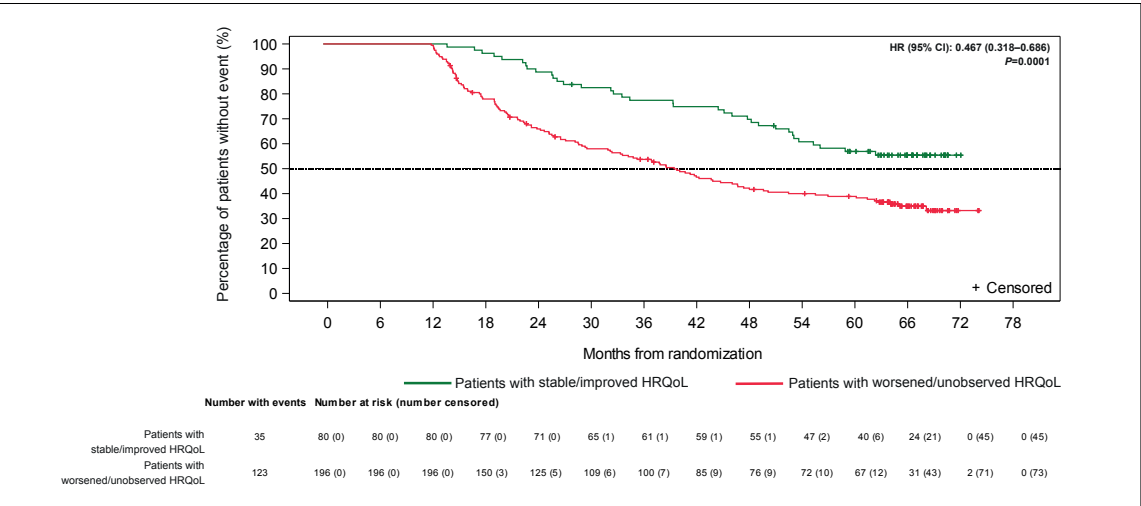

Supplement: oyae003_suppl_Supplementary_Figure_S2 [file oyae003_suppl_supplementary_figure_s2.pdf]
