# Supplementary material for: The Relationship Between Health-Related Quality of Life and Overall Survival in Patients With Advanced Renal Cell Carcinoma in CheckMate 214
Source: Oncologist. 2024 Jan 27;29(6):511–8. doi: 10.1093/oncolo/oyae003 (PMC11144972; doi:10.1093/oncolo/oyae003)
Supplement: oyae003_suppl_Supplementary_Figure_Captions [file oyae003_suppl_supplementary_figure_captions.docx]

**Supplementary material**

**Figure S1**. Kaplan–Meier plots of OS by response in FKSI-19 total score at different landmarks (overall [threshold=5]): patients with intermediate- or poor-risk RCC (all randomized)^a^

1. 6-month landmark

B. 9-month landmark

C.12-month landmark

^a^HR is relative to patients with worsening or unobserved HRQoL, with HR < 1 favoring patients with stable or improved HRQoL. HR is derived from a stratified Cox regression model with response as the only covariate and strata as the randomization factors. The *P* value corresponds to the Cox regression model.

**Figure S2**. Kaplan–Meier plots of OS by response in FKSI-19 total score at different landmarks for each treatment group (threshold=5): patients with intermediate- or poor-risk RCC (all randomized)^a^

A. 6-month landmark for nivolumab + ipilimumab

B. 6-month landmark for sunitinib alone

C. 9-month landmark for nivolumab + ipilimumab

D. 9-month landmark for sunitinib alone

E. 12-month landmark for nivolumab + ipilimumab

F. 12-month landmark for sunitinib alone

^a^HR is relative to patients with a worsening or unobserved HRQoL response, with HR < 1 favoring patients with stable or improved HRQoL. HR is derived from a stratified Cox regression model with response as the only covariate and strata as the randomization factors. The *P* value corresponds to the Cox regression model.
